# Infections due to *Cellulosimicrobium* species: case report and literature review

**DOI:** 10.1186/s12879-019-4440-2

**Published:** 2019-09-18

**Authors:** María Rivero, Javier Alonso, María Fernanda Ramón, Nancy Gonzales, Ana Pozo, Itxaso Marín, Ana Navascués, Regina Juanbeltz

**Affiliations:** 1 Infectious Diseases Unit, Complejo Hospitalario de Navarra, Calle Irunlarrea 3, 31008 Pamplona, Navarra Spain; 2 Navarre Institute for Health Research (IdiSNA), Calle Irunlarrea 3, 31008 Pamplona, Navarra Spain; 3 Geriatric Department, Complejo Hospitalario de Navarra, Calle Irunlarrea 3, 31008 Pamplona, Navarra Spain; 4 Service of Clinical Microbiology, Complejo Hospitalario de Navarra, Calle Irunlarrea 3, 31008 Pamplona, Navarra Spain; 5 Instituto de Salud Pública de Navarra, Calle Leyre 15, 31003 Pamplona, Navarra Spain; 6 CIBER Epidemiología y Salud Pública (CIBERESP), Instituto de Salud Carlos III, Calle Monforte de Lemos 3-5, Pabellón 11, Planta 0, 28029 Madrid, Spain

**Keywords:** *Cellulosimicrobium*, *Oerskovia*, Central venous catheter, Endocarditis, Foreign body

## Abstract

**Background:**

*Cellulosimicrobium* species, formely known as *Oerskovia* species, are gram-positive bacilli belonging to the order *Actinomycetales*. They rarely cause human infections. The genus comprises two pathogenic species in humans: *C. cellulans* and *C. funkei*. Based on a case report, we provide a review of the literature of infections caused by *Cellulosimicrobium*/*Oerskovia*, in order to improve our knowledge of this unusual infection.

**Case presentation:**

An 82-year-old woman with aortic prosthetic valve presented to the hospital with fever and heart failure. Further work up revealed the diagnosis of *C. cellulans* infective endocarditis (IE). The strain was identified by MALDI-TOF MS, API Coryne and 16S rRNA sequencing. The patient was deemed not to be an operative candidate and died despite the antibiotic therapy 35 days after admission.

**Conclusions:**

Reviewing cases of *Cellulosimicrobium* species infections and communicating the successful and unsuccessful clinical experiences can assist future healthcare providers. Our case and those previously reported indicate that *Cellulosimicrobium* species usually infect immunocompromised patients or foreign body carriers. The most frequent pattern of infection is central venous catheter related bacteremia. The optimal treatment should include foreign body removal and valve surgery should be considered in case of IE.

## Background

*Cellulosimicrobium* species are gram-positive bacilli belonging to the order *Actinomycetales*. Formerly known as *Oerskovia,* they were reclassified in 2001 as *Cellulosimicrobium* by Schumann et al. [1]. The genus comprises various species [2], but only two have been described as pathogens in humans: *C. cellulans*, formerly known as *Oerskovia xanthineolytica*, and *C. funkei*, formerly *Oerskovia turbata*. They are widely distributed in the environment and have been isolated mainly from soil, water, and grass cuttings [3]. Despite this ubiquitous distribution, *Cellulosimicrobium* species rarely cause infections in humans. Here, we report a case of prosthetic infective endocarditis (IE) caused by *C. cellulans* and present a complete literature review of infections caused by these organisms, in order to improve our knowledge of this unusual infection.

## Case presentation

An 82-year-old woman with type 2 diabetes mellitus and chronic renal failure was admitted to the hospital with a 7-day history of fever, delirium, and dyspnea. She had undergone an aortic valve replacement (Perceval sutureless bioprosthesis) 18 months prior due to aortic stenosis. The immediate post cardiac surgery period was complicated by paroxysmal atrial fibrillation, transudative left-sided pleural effusion, and oligoanuric renal failure. She did not present any infectious complications and the median sternotomy incision closed normally. Between 1 and 14 months after aortic surgery, she was admitted to the hospital five times because of severe clinical heart failure of unclear cause and some episode of paroxysmal atrial fibrillation. No fever or other signs of infection were detected at all this time, and did not receive any antibiotic treatment. A transesophageal echocardiogram performed 3 months after surgery showed an aortic prosthesis without alterations. On physical examination, her temperature was 39 °C, she was confused and tachypneic. A 3/6 systolic ejection murmur in the aortic position and basal crackles were identified. She presented grade II uninfected pressure ulcers on heels and sacrococcygeal region. Laboratory tests showed a normal blood cell count, a serum creatinine of 2.14 mg/dL, and an increased C-reactive protein (13 mg/dL) and hyperglycemia (628 mg/dL). A chest X-ray showed bilateral pleural effusion and interstitial pulmonary edema. Two sets of aerobic and anaerobic blood culture bottles were drawn at admission, and empiric ceftriaxone (2 g daily) and levofloxacin adjusted to renal function (250 mg daily, intravenous) were started. After 26 to 80 h of incubation into the BACTEC FX system (Becton, Dickinson and Company), all four blood culture bottles were positive. Gram stain showed coryneform gram-positive bacilli with occasional branching forms. After incubation on CNA agar and chocolate agar, colonies were less than 2 mm in size, glistening and yellow. The colonies penetrated into the agar upon further incubation. On the 5th day of admission, blood cultures were again obtained, and the same organism grew in 1 of the 4 bottles. The isolates were initially identified by matrix-assisted laser desorption ionization-time of flight mass spectrometry (MALDI-TOF MS, Bruker Daltonics) as *C. cellulans*. Thereafter, the identification was confirmed by API Coryne strip (bioMérieux; code number 7572767), which was an “excellent identification” for *C. cellulans* with a reliability of 99.9%, and by sequencing the 16S rRNA (using the BLAST Sequence Analysis Tool of GenBank database), showing a 100% similarity with *C. cellulans* and 99.8% with *C. funkei*. Antimicrobial susceptibility tests were performed using a microdilution microtiter panel MICroSTREP plus 6 (MicroScan Walk Away, Beckman Coulter). Following EUCAST breakpoints criteria for *Corynebacterium*, the isolate was susceptible or presumably susceptible (for antibiotics without EUCAST breakpoints, but with low MIC) to amoxicillin-clavulanate (MIC = 2 mg/L), daptomycin (MIC = 0.5 mg/L), levofloxacin (MIC = 2 mg/L), linezolid (MIC≤1 mg/L), tetracycline (MIC≤1 mg/L), trimethoprim-sulfamethoxazole (MIC = 0.006 mg/L) and vancomycin (MIC = 0.5 mg/L), and resistant or presumably resistant to amikacin (MIC = 32 mg/L), cefotaxime (MIC> 2 mg/L), ciprofloxacin (MIC> 2 mg/L), clindamycin (MIC> 2 mg/L), erythromycin (MIC = 1 mg/L), gentamycin (MIC = 4 mg/L), imipenem (MIC = 4 mg/L), meropenem (MIC = 8 mg/L) and rifampin (MIC = 1 mg/L). The MICs of amoxicillin-clavulanate, cefotaxime, meropenem, trimethoprim-sulfamethoxazole and vancomycin were also determined by Etest® (bioMérieux) using Mueller-Hinton agar plus 5% blood, and similar results were found.

On the 7th day, a transthoracic echocardiogram did not show alterations. Therapy was switched to amoxicillin-clavulanate (1 g three times daily, intravenous), and new blood cultures obtained 24 h later were negative. A transesophageal echocardiogram performed on the 9th day of stay revealed an echogenic and mobile vegetation of 6 × 9 mm on the prosthetic aortic valve attached to the commissure between the right coronary cusp and the non-coronary cusp. Prosthetic valve function was otherwise normal. On the 11th day, amoxicillin-clavulanate was switched to vancomycin adjusted to renal function (750 mg daily) plus linezolid (600 mg twice daily, intravenous). Surgical replacement was considered inappropriate in this patient due to comorbidity, advanced age, limited mobility and family rejection. In the following days, she developed severe anemia, acute confusional state and refractory heart failure. End-of-life decision-making was implemented, prioritizing symptom control, and antibiotic therapy was switched back to amoxicillin-clavulanate on day 22nd of admission. On the 28th day, she was discharged to another hospital for palliative care, dying 7 days later because of sepsis and severe heart failure. A postmortem examination was not performed.

## Method

We searched MEDLINE using the following keywords: “*Cellulosimicrobium*”, “*C. cellulans*”, “*C. funkei”*, “*Cellulosimicrobium* species”, “*Oerskovia”*, “*O. xanthineolytica”*, “*O. turbata”*, and “*Oerskovia* species”. The search period was from 1970, the year in which the *Oerskovia* genus was first described [4], until March 2019. The reference lists of identified articles were also reviewed to find additional cases. No language restrictions were applied. We excluded from this analysis one of the cases published by Funke and Marty [5] because the authors interpreted isolation as a contaminant, as well as a series of 35 clinical isolates of *Oerskovia* species [6] because the corresponding clinical data was not available.

## Discussion and conclusions

*Cellulosimicrobium* species are gram-positive bacilli belonging to the order *Actinomycetales*, suborder *Micrococcineae*, family *Promicromonosporaceae*. They are organisms that have undergone several taxonomic changes. *Oerskovia xanthineolytica*, described in 1972 [3], was reclassified in 1982 as *Cellulomonas cellulans* [7]. In 2001, Schumann et al. [1] proposed its reclassification in a new genus, *Cellulosimicrobium*, and *Oerskovia xanthineolytica* was renamed as *Cellulosimicrobium cellulans*. The rationale for this proposal was the distinct position of this species on the neighbour-joining phylogenetic tree, based on 16S rRNA gene sequencing, and the presence of unique peptidoglycan in the cell wall, which was absent from authentic *Cellulomonas* species. The genus comprised only the type species *C. cellulans* until 2006, when Brown et al. [8] proposed that some clinical isolates identified in 1970 as *Oerskovia turbata* [4] be included in this genus as a new species, *Cellulosimicrobium funkei*.

A total of 43 cases of infections caused by *Cellulosimicrobium* (or *Oerskovia*) were identified in the literature review. They are summarized in Table 1 [5, 9–47]. There were 25 (58%) males, and the median age was 49 years (range 0–81 years). Seven (16%) were children. Forty cases were sporadic infections and 3 were associated with an outbreak of endophthalmitis after cataract surgery at a hospital in Turkey [42]. Most of the cases were from the USA (*n* = 18) and Spain (*n* = 6), and almost half were published in the last 15 years. Twenty-six (60%) patients suffered from a variety of chronic underlying illnesses that involved immune dysfunction. The most frequent were chronic kidney failure (8 cases, 5 of them on peritoneal dialysis, 1 on hemodialysis, and 1 a renal transplant patient), diabetes mellitus (4 cases), solid organ neoplasia (4 cases), neutropenia (4 cases), hematologic malignancies (3 cases, 2 of them with bone marrow transplant), HIV infection (3 cases), alcoholism (2 cases), and inflammatory bowel disease (2 cases).

**Table 1:** Reported cases of *Cellulosimicrobium* species (or *Oerskovia*) infections

Ref	Age/sex	Predisposing condition or underlying disease	Species (specimen)	Type of infection	Foreign body (time before)/ removal	Antibiotic therapy^a^ and surgery	Failed antibiotic therapy alone^b^	Outcome
[9]	68 yr./M	Aortic insufficiency, prosthetic valve, Crohn’s, ankylosing spondylitis, steroids	*O. turbata* (blood, prosthetic heart valve)	Endocarditis	Prosthetic heart valve (2 mo)/yes	PEN, AMP + ERY, SXT, SXT + AMP, SXT + AMX Aortic valve replacement	Yes	Cure
[10]	3 yr./M	Acute myelogenous leukemia, neutropenia	*O. turbata* (blood)	CVC-related bacteremia	CVC (1 mo)/yes	AMK	Yes	Cure
[11]	40 yr./F	Crohn’s, short bowel syndrome	*Oerskovia* species (blood, TPN solution)	CVC-related bacteremia	CVC (9 mo)/no	VAN+GEN + MET, VAN	No	Cure
[12]	40 yr./M	Alcoholism, cirrhosis	*O. xanthineolytica* (blood)	Bacteremia	None	VAN+GEN + CLI	NA	Cure
[13]	54 yr./F	Metastatic breast cancer, chemotherapy	*O. xanthineolytica* (blood)	CVC-related? bacteremia	CVC (4 mo) (unclear role)/no	CXM, VAN	No	Cure
[14]	49 yr./F	Metastatic colonic cancer	*O. xanthineolytica* (blood, CVC tip)	CVC-related bacteremia	CVC (15 mo)/no	VAN	No	Cure
[15]	27 yr./M	AIDS, neutropenia	*O. turbata* (blood)	CVC-related bacteremia	CVC (4 wk)/yes	IMP+AMK	NA	Cure
[16]	53 yr./F	Non-Hodgkin’s lymphoma, BMT, neutropenia	*O. xanthineolytica* (blood, CVC tip)	CVC-related bacteremia ➔Endocarditis	CVC (1 yr)/yes	DOX, CLI, MPM, AMX + SXT, IMP+AMK, CLA + RIF, PEN	NA	Death
[17]	64 yr./F	Peritonitis carcinomatosa	*O. xanthineolytica* (blood)	Bacteremia	None	PTZ, PTZ + NET, VAN+NET, PTZ + NET	NA	Cure
[18]	31 yr./M	CRF, renal transplant, DM	*O. xanthineolytica* (blood, heart valve)	CVC-related bacteremia ➔Endocarditis	CVC (NR)/yes	AMS, AMS + VAN, CAZ + VAN, VAN Aortic valve replacement	NA	Cure
[19]	13 yr./M	Short bowel syndrome	*C. cellulans* (blood)	CVC-related bacteremia	CVC (NR)/no	VAN+CTX, VAN+GEN, VAN, VAN+RIF	No	Cure
[20]	1 d/M	None	*C. cellulans* (blood)	Bacteremia	None	CTX + AMP, VAN	NA	Cure
[21]	81 yr./M	Aortic stenosis, prosthetic valve	*C. funkei* (blood)	Endocarditis	Prosthetic heart valve (7 mo)/no	AMC, VAN+GEN	Yes	Death
[22]	59 yr./M	COPD, arteriosclerosis	*C. cellulans* (blood)	CVC-related bacteremia	CVC (7 d)/yes	VAN+PTZ	NA	Death
[23]	80 yr./F	CRF, hemodialysis	*C. cellulans* (blood)	CVC-related bacteremia	CVC (NR)/yes	VAN	Yes	Cure^*c*^
[24]	59 yr./F	Metastatic rectal cancer	*C. cellulans* (blood)	CVC-related bacteremia	CVC (NR)/yes	VAN, VAN+IMP, VAN	Yes	Cure
[25]	44 yr./F	T-cell lymphoma, BMT, neutropenia	*Cellulosimicrobium* species (blood, CVC tip)	CVC-related bacteremia	CVC (5 wk)/yes	VAN+CPM, VAN+MPM, VAN	NA	Death
[26]	≈50 yr./M	DM	*Oerskovia* species (blood)	Osteomyelitis	None	VAN+CPM + MET, SXT Amputation	NA	Cure
[27]	47 yr./F	Kidney trouble?	*Oerskovia* species (kidney)	Pyonephrosis	None	Nephrectomy	NA	Cure
[28]	47 yr./M	Penetrating eye injury, steroids	*O. xanthineolytica* (vitreous humor)	Endophthalmitis	Intraocular metallic object (12 d)/yes	GEN (sc), PEN, PEN+CFL Vitrectomy	NA	Cure
[29]	38 yr./F	Hydrocephalus	*O. xanthineolytica* (CSF)	Meningitis	VP shunt (7 yr)/yes	PEN, PEN+RIF	Yes	Cure
[30]	70 yr./M	CRF, CAPD	*O. xanthineolytica* (peritoneal fluid, PC tip)	Peritonitis	PC (11 yr)/yes	VAN+GEN	Yes	Cure
[31]	23 yr./M	AIDS	*O. turbata* (subcutaneous fluid)	Soft tissue infection	None	Debridement	NA	Cure
[5]	53 yr./F	Arthropathy, intramuscular injections	*O. xanthineolytica* (subcutaneous fluid)	Soft tissue infection	None	DOX	NA	Cure
[5]	72 yr./M	None	*O. xanthineolytica* (bile)	Cholecystitis	None	CFX Cholecystectomy	NA	Cure
[32]	59 yr./F	CRF, CAPD, DM	*O. xanthineolytica* (peritoneal fluid)	Peritonitis	PC (6 wk)/no	VAN (ip) + TOB, DOX	No	Cure
[33]	28 yr./F	None	*O. xanthineolytica* (cornea)	Keratitis	Contact lens (NR)/yes	CFZ (drops) + GEN (drops)	NA	Cure
[34]	72 yr./M	Gonarthrosis, knee prosthesis, closed knee injury, alcoholism	*O. xanthineolytica* (sinovial tissue, bone)	Prosthetic joint infection	Prosthetic joint (3 yr)/yes	VAN, SXT Two-stage reimplantation	NA	Cure
[35]	13 yr./F	CRF, CAPD	*O. xanthineolytica* (peritoneal fluid)	Peritonitis	PC (11 mo)/no	VAN (ip)	No	Cure
[36]	23 mo/F	None	*O. xanthineolytica* (intervertebral biopsy)	Spondylodiscitis	None	CFT + RIF, CXM + RIF	NA	Cure
[37]	48 yr./M	HIV	*C. cellulans* (ulcer)	Tongue ulcer	None	PEN+AZI	NA	Cure
[38]	44 yr./M	Hydrocephalus	*O. xanthineolytica* (CSF, VP shunt)	Meningitis	VP shunt (3 mo)/yes	VAN+RIF	Yes	Cure
[39]	76 yr./M	COPD	*O. turbata* (bile)	Cholecystitis	None	Cholecystectomy	NA	Cure
[40]	5 yr./M	Penetrating hand injury	*C. cellulans* (tendon, deep biopsy)	Tenosynovitis	Splinters (4 mo)/yes	SXT + RIF Debridement	NA	Cure
[41]	1 d/M	Premature	*O. xanthineolytica* (lung)	Pneumonia	None	CTX + GEN	NA	Death
[42]	72 yr./F	Cataract surgery	*C. cellulans* (vitreous humor)	Endophthalmitis	Intraocular lens (1 d)/no	VAN (iv) + CAZ (iv)	No	Cure
[42]	74 yr./M	Cataract surgery, arteriosclerosis	*C. cellulans* (vitreous humor)	Endophthalmitis	Intraocular lens (3 d)/no	VAN (iv) + CAZ (iv)	No	Cure
[42]	78 yr./F	Cataract surgery	*C. cellulans* (vitreous humor)	Endophthalmitis	Intraocular lens (7 d)/no	VAN (iv) + CAZ (iv) Vitrectomy	No	Cure
[43]	28 yr./M	Penetrating eye injury	*C. cellulans* (vitreous humor)	Endophthalmitis	Intraocular metallic object (2 d)/yes	VAN+CAZ + CLI, VAN+CAZ + MOX (all iv) Vitrectomy	Yes	Cure
[44]	45 yr./M	None	*C. cellulans* (vitreous humor)	Endophthalmitis	None	CIP, VAN (iv) + CAZ (iv) Vitrectomy	NA	Cure
[45]	62 yr./M	CRF, CAPD	*O. turbata* (peritoneal fluid)	Peritonitis	PC (3 yr)/yes	VAN (ip) + GEN (ip) + CIP, VAN (ip) + SXT, VAN (ip) + GEN (ip) + SXT, VAN (ip)	Yes	Cure
[46]	81 yr./M	CRF, penetrating knee injury	*C. cellulans* (sinovial fluid)	Arthritis	None	LEV, LEV+LNZ, LNZ + RIF Debridement	NA	Cure
[47]	50 yr./F	CRF, CAPD, DM	*C. cellulans* (peritoneal fluid, PC tip)	Peritonitis	PC (15 mo)/yes	CAZ (ip) + TOB (ip), CAZ (ip) + TOB (ip) + VAN (ip), VAN	Yes	Cure

*AIDS* Acquired immunodeficiency síndrome, *AMC* Amoxicillin-clavulanate, *AMK* Amikacin, *AMP* Ampicillin, *AMS* Ampicilllin-sulbactam, *AMX* Amoxicillin, *AZI* Azithromycin, *BMT* Bone marrow transplant, *CAPD* Chronic ambulatory peritoneal dialysis, *CAZ* Ceftazidime, *CFL* Cephalexin, *CFT* Ceftriaxone, *CFX* Cefoxitin, *CFZ* Cefazolin, *CIP* Ciprofloxacin, *CLA* Clarithromycin, *CLI* Clindamycin, *COPD* Chronic obstructive pulmonary disease, *CPM* Cefepime, *CRF* Chronic renal failure, *CSF* Cerebrospinal fluid, *CTX* Cefotaxime, *CVC* Central venous catheter, *CXM* Cefuroxime, *DM* Diabetes mellitus, *DOX* Doxycycline, *ERY* Erythromycin, *GEN* Gentamycin, *HIV* Human immunodeficiency virus, *IMP* Imipenem, *ip* Intraperitoneal, *iv* Intravitreal, *LEV* Levofloxacin, *LNZ* Linezolid, *MET* Metronidazole, *MOX* Moxifloxacin, *MPM* Meropenem, *NA* Not applicable, *NR* Not reported, *NET* Netilmicin, *PC* Peritoneal catheter, *PEN* Penicillin, *PR* Present report, *PTZ* Piperacillin-tazobactam, *RIF* Rifampin, *sc* Subconjunctival, *SXT* Trimetroprim-sulfamethoxazole, *TOB* Tobramycin, *TPN* Total parenteral nutrition, *VAN* Vancomycin, *VP* Ventriculoperitoneal

^a^The route of administration is systemic if it is not specified

^b^That is, foreign body associated infections which were not cured by antibiotic therapy alone (10 cases requiring its removal and another case resulting in death)

^c^Later death not attributable to the infection

Infection was related to the presence of medical devices or foreign bodies in 29 (67%) cases, mainly with central venous catheter (CVC) (*n* = 12) causing CVC-related bacteremia, but also with peritoneal catheters, intraocular lenses, cardiac valve or joint prostheses, or ventriculo-peritoneal shunts. Moreover, spontaneous infections have also been reported, including primary bacteremia, pneumonia, cholecystitis, pyonephrosis, endophthalmitis, soft tissue infection, arthritis and osteomyelitis. Of all the 43 cases, 7 (16%) were either non-immunocompromised patients or non-carriers of foreign bodies. Our review shows 4 previous cases of IE corresponded to 2 cases with early prosthetic aortic IE by the currently named *C. funkei* (one of them as a result of implanting a contaminated prosthesis) diagnosed respectively 2 and 7 months after cardiac surgery, and 2 cases with native IE by the current *C. cellulans* (both secondary to CVC-related bacteremia). Thus, the patient presented herein is the first with prosthetic IE due to *C. cellulans*. The pathogenesis of the infection in our patient can be debatable. After cardiac surgery, she presented a torpid evolution with several hospital admissions, but had no infectious complications related to any medical procedure, signs of cutaneous infection in pressure ulcers and the onset of symptoms was not associated to any possible inoculation procedure. Without being able to completely exclude a hematogenous spread to the prosthetic valve, we believe that in the absence of another source of infection it is more reasonable to think of a late presentation (or late diagnosis) of a periprosthetic valve infection. Many of *Actinomycetales* infections are characterized by a relatively long history with minimal clinical signs of infection at initial presentation, which often leads to a delay in diagnosis. The diagnosis of IE in our patient was based on the Duke clinical criteria, as no pathological specimens were available. Bacterial identification was performed by MALDI-TOF MS and was confirmed at least to the genus level by the API Coryne system and the 16S rRNA sequencing. They are reliable techniques that have been used for the bacterial identification in many of the reported cases [5, 13, 15–17, 19–25, 34, 35, 37, 40–42, 44–47]. However, the API Coryne system and the 16S rRNA sequencing may not be able to distinguish between the two species with absolute certainty [21]. As these organisms can very easily be confused by its appearance with *Corynebacterium* species, they can be disregarded as contaminants if microbiologic identification is incomplete. A full microbiological identification must be attempted at least to the genus level when coryneform gram-positive bacilli are isolated in this patient population.

Given the rarity of this infection, there are no standardized recommendations for the treatment of infections caused by *Cellulosimicrobium* species. It is difficult to draw conclusions from the reviewed cases concerning the real effectiveness of the antibiotic regimens because the antimicrobial agents used were not homogeneous, the duration of therapy varied and was not always specified, and as in our case there were frequent changes in antibiotic therapy in many of them. Table 2 shows the antimicrobial susceptibility data of *Cellulosimicrobium* species, taken from the cases reviewed. Based on these in vitro results, vancomycin and linezolid would be considered the drugs of choice. However, this should be interpreted with caution due to the fact that there are no standardized methods or interpretation breakpoints for this organism. Thus, the methods used and the interpretation criteria, data available in 19 of the reviewed cases, were variable (mainly disk diffusion and microdilution). Antibiotic therapy was administered to 40 patients. Twenty-six patients were treated with vancomycin, either as single-agent therapy (*n* = 6) or in combination therapy (*n* = 20), and as first-line therapy (*n* = 19) or as follow-up therapy (*n* = 7). We observed that the mortality rate was similar between patients with or without vancomycin on the antibiotic regimen. Only one patient was treated with linezolid and it is not possible to assess the clinical efficacy of this drug. There is no clinical experience nor data on their in vitro activity in the cases reviewed in this study with more recent antibiotics such as daptomycin or the new glycopeptides. In our case, it was presumed that the isolated bacteria was susceptible in vitro to daptomycin. The broad spectrum of activity of these ‘new’ antibiotics against gram-positive bacteria, including different *Actinobacteria*, suggests that these antibiotics could be a therapeutic alternative.

**Table 2:** Antibiotic susceptibility data of *Cellulosimicrobium* species (or *Oerskovia*) in reported cases

	All species			*C. cellulans*		*C. funkei*	
	No. of isolates	MIC range (mg/L)	% Susceptibility	No. of isolates	% Susceptibility	No. of isolates	% Susceptibility
AMC	4	1/0.5–16/8	50%	4	50%	–	–
AMK	11	4–16	73%	7	57%	3	100%
CFZ	9	1–8	67%	8	75%	–	–
CIP	15	1 - > 8	13%	12	17%	0	0%
CLI	13	2–8	8%	12	0%	–	–
CTX	7	8–64	29%	5	20%	2	50%
ERY	19	1–16	26%	13	15%	4	50%
GEN	14	2–16	43%	9	11%	4	100%
IMP	8	< 0.25 - > 16	75%	6	83%	2	50%
LNZ	4	0.5–1	100%	4	100%	–	–
PEN	24	0.012–4	25%	17	18%	4	25%
RIF	12	< 0.5–4	75%	9	78%	3	67%
SXT	20	< 0.06/1.19–8/152	85%	15	93%	4	50%
TET	16	1–8	50%	13	54%	2	50%
VAN	30	≤ 0.25 - < 4	100%	23	100%	5	100%

*AMC* Amoxicillin-clavulanate, *AMK* Amikacin, *CFZ* Cefazolin, *CIP* Ciprofloxacin, *CLI* Clindamycin, *CTX* Cefotaxime, *ERY* Erythromycin, *GEN* Gentamycin, *IMP* Imipenem, *LNZ* Linezolid,

*MIC* Minimum inhibitory concentration, *PEN* Penicillin, *RIF* Rifampin, *SXT* Trimetroprim-sulfamethoxazole, *TET* Tetracycline, *VAN* Vancomycin

In addition to antibiotic therapy, debridement of infected tissue in localized infections and foreign bodies removal in the infections associated with them were usually required for complete recovery. Nineteen of the 29 patients with foreign body associated infections were treated with foreign body removal and 16 achieved cure. This includes 10 patients with persistence or recurrence of infection despite the use of active antibiotic therapy until the foreign body was finally removed. In all 10 patients treated without foreign body removal, the antibiotic regimen included vancomycin, and a recovery with complete eradication of the pathogen was obtained in 9 patients. Thus, treatment with antibiotics alone failed in 38% (11/29) of the foreign body associated infections [9, 10, 21, 23, 24, 29, 30, 38, 43, 45, 47].

Five patients died: a premature patient with pneumonia who was treated with antibiotics without in vitro activity against *O. xanthineolytica*, 2 patients with CVC-related bacteremia, and 2 patients with non-operated IE. According to this review, the consideration of *Cellulosimicrobium* as a relatively avirulent bacteria [19, 34] is only true in localized infections. The mortality rate in patients with disseminated infection, particularly in patients with IE, is high. Of the 4 previous cases with IE, 2 patients treated without cardiac surgery died and the 2 patients who underwent valve surgery survived. In our patient, surgical replacement was not considered adequate due to family rejection, and also due to comorbidity and advanced age. However, the data seem to indicate that surgical treatment of IE caused by *Cellulosimicrobium* species is necessary for cure, placing it among the infecting organisms with indication of IE surgery.

Following the updated taxonomy, the identified species in the cases reviewed was *C. cellulans* in 32 (74%) cases and *C. funkei* in 7 (16%) cases. Species identification was not reached in 4 cases. We did not find correlation between the site of infection, baseline characteristics of patients, response to treatment, outcome, and the infecting species. As Table 2 shows the identified susceptibility of both species to vancomycin was 100%. *C. funkei* was more susceptible than *C. cellulans* to gentamicin (100% vs. 11%), whereas *C. cellulans* was more susceptible than *C. funkei* to trimethoprim-sulfamethoxazole (93% vs. 50%). No differences in susceptibility to imipenem were detected between the two species in the reviewed cases. Brown et al. [8] reported that the two *Cellulosimicrobium* species were resistant to aminoglycosides. Among its main phenotypic differences, they found that *C. funkei*, unlike *C. cellulans*, was susceptible to imipenem and resistant to trimethoprim-sulfamethoxazole. In this review, we found that *C. funkei*, unlike *C. cellulans*, was susceptible to gentamicin and that there were no significant differences in imipenem susceptibility between both species. It has already been suggested previously that susceptibility to imipenem might not be different between both species [21], and according to this review, susceptibility to aminoglycosides may be a reliable phenotypic test for differentiating *C. funkei* from *C. cellulans*.

In conclusion, the uncommon *Cellulosimicrobium* infection usually occurs in immunocompromised hosts or in patients with medical devices or foreign bodies that compromise the integrity of defensive mechanisms. The most frequent pattern of *Cellulosimicrobium* infection is CVC-related bacteremia. The optimal treatment should include the withdrawal of the foreign body. If this is not possible, vancomycin should probably be part of the antibiotic regimen. Valve surgery should be considered in native or prosthetic *Cellulosimicrobium* IE because it probably improves the outcome. We should be aware of this opportunistic pathogen, as it is likely that there will be an increase in its prevalence, related to the high survival rate of immunocompromised patients, the increasing use of long-term medical devices, and the advances in microbiological diagnostic techniques.

## Data Availability

All data generated or analysed during this study are included in this published article.

## Abbreviations

CVC: Central venous catheter
IE: Infective endocarditis;
MALDI-TOF MS: Matrix-assisted laser desorption ionization-time of flight mass spectrometry
MIC: Minimum inhibitory concentration

## References

[1] Schumann P, Weiss N, Stackebrandt E (2001). Reclassification of Cellulomonas cellulans (Stackebrandt and Keddie 1986) as Cellulosimicrobium cellulans gen. Nov., comb. nov. Int J Syst Evol Microbiol.

[2] Oh M, Kim JH, Yoon JH, Schumann P, Kim W (2018). Cellulosimicrobium arenosum sp. Nov., isolated from marine sediment sand. Curr Microbiol.

[3] Lechevalier MP (1972). Description of a new species, Oerskovia xanthineolytica, and emendation of Oerskovia Prauser et al. Int J Syst Bacteriol.

[4] Prauser H, Lechevalier MP, Lechevalier H (1970). Description of Oerskovia gen n to harbor Orskov's motile nocardia. Appl Microbiol.

[5] Funke G, Marty N (1995). Three strains of Oerskovia xanthineolytica from clinical specimens. Clin Microbiol Newsl.

[6] Sottnek FO, Brown JM, Weaver RE, Caroll GF (1977). Recognition of *Oerskovia* species in the clinical laboratory: characterization of 35 isolates. Int J Syst Bacteriol.

[7] Stackebrandt E, Seiler H, Schleifer KH (1982). Union of the genera *Cellulomonas* Bergey *et al*. and *Oerskovia* Prauser *et al*. in a redefined genus. Zentbl Bakteriol Parasitenkd Infektionskr Hyg Abt 1 Orig.

[8] Brown JM, Steigerwalt AG, Morey RE, Daneshvar MI, Romero LJ, McNeil MM (2006). Characterization of clinical isolates previously identified as Oerskovia turbata: proposal of Cellulosimicrobium funkei sp. nov. and emended description of the genus Cellulosimicrobium. Int J Syst Evol Microbiol.

[9] Reller LB, Maddoux GL, Eckman MR, Pappas G (1975). Bacterial endocarditis caused by Oerskovia turbata. Ann Intern Med.

[10] LeProwse CR, McNeil MM, McCarty JM (1989). Catheter-related bacteremia caused by Oerskovia turbata. J Clin Microbiol.

[11] Guss WJ, Ament ME (1989). Oerskovia infection caused by contaminated home parenteral nutrition solution. Arch Intern Med.

[12] Truant AL, Satishchandran V, Eisenstaedt R, Richman P, McNeil MM (1992). Oerskovia xanthineolytica and methicillin-resistant Staphylococcus aureus in a patient with cirrhosis and variceal hemorrhage. Eur J Clin Microbiol Infect Dis.

[13] McDonald CL, Chapin-Robertson K, Dill SR, Martino RL (1994). Oerskovia xanthineolytica bacteremia in an immunocompromised patient with pneumonia. Diagn Microbiol Infect Dis.

[14] Maguire JD, McCarthy MC, Decker CF (1996). Oerskovia xanthineolytica bacteremia in an immunocompromised host: case report and review. Clin Infect Dis.

[15] Lair MI, Bentolila S, Grenet D, Cahen P, Honderlick P (1996). Oerskovia turbata and Comamonas acidovorans bacteremia in a patient with AIDS. Eur J Clin Microbiol Infect Dis.

[16] Ellerbroek P, Kuipers S, Rozenberg-Arska M, Verdonck LF, Petersen EJ (1998). Oerskovia xanthineolytica: a new pathogen in bone marrow transplantation. Bone Marrow Transplant.

[17] Niamut SM, van der Vorm ER, van Luyn-Wiegers CG, Gökemeijer JD (2003). Oerskovia xanthineolytica bacteremia in an immunocompromised patient without a foreign body. Eur J Clin Microbiol Infect Dis.

[18] Urbina BY, Gohh R, Fischer SA (2003). Oerskovia xanthineolytica endocarditis in a renal transplant patient: case report and review of the literature. Transpl Infect Dis.

[19] Rowlinson MC, Bruckner DA, Hinnebusch C, Nielsen K, Deville JG (2006). Clearance of Cellulosimicrobium cellulans bacteremia in a child without central venous catheter removal. J Clin Microbiol.

[20] Casanova-Román M, Sanchez-Porto A, Gomar JL, Casanova-Bellido M (2010). Early-onset neonatal sepsis due to Cellulosimicrobium cellulans. Infection..

[21] Petkar H, Li A, Bunce N, Duffy K, Malnick H, Shah JJ (2011). Cellulosimicrobium funkei: first report of infection in a nonimmunocompromised patient and useful phenotypic tests for differentiation from Cellulosimicrobium cellulans and Cellulosimicrobium terreum. J Clin Microbiol.

[22] Delport J, Tamiko A, Venkatesh R, Lannigan R, John M, McCormick JK. Cellulosimicrobium cellulans isolated from a patient with acute renal failure. JMM Case Rep. 2014. 10.1099/jmmcr.0.000976.

[23] Coletta-Griborio E, Rodriguez Portela G, Núñez García JM, Bratos-Pérez MÁ (2017). Bacteremia due to Cellulosimicrobium cellulans associated with central catheter for hemodialysis. Enferm Infecc Microbiol Clin.

[24] Ponce-Alonso M, Del Campo R, Fortun J, Cantón R, Morosini MI (2017). First description of late recurrence of catheter-associated bacteraemia due to Cellulosimicrobium cellulans. Enferm Infecc Microbiol Clin.

[25] Gonzales Zamora JA, Camps N (2018). Bacteremia caused by cellulosimicrobium in a bone marrow transplant patient: a case report and literature review. IDCases..

[26] Oikonomou KG, Mcwilliams CS, Moussa MM (2018). *Oerskovia* species bacteremia in a diabetic patient. J Global Infect Dis.

[27] Cruickshank JG, Gawler AH, Shaldon C (1979). Oerskovia species: rare opportunistic pathogens. J Med Microbiol.

[28] Hussain Z, Gonder JR, Lannigan R, Stoakes L (1987). Endophthalmitis due to Oerskovia xanthineolytica. Can J Ophthalmol.

[29] Kailath EJ, Goldstein E, Wagner FH (1988). Meningitis caused by Oerskovia xanthineolytica. Am J Med Sci.

[30] Rihs JD, McNeil MM, Brown JM, Yu VL (1990). Oerskovia xanthineolytica implicated in peritonitis associated with peritoneal dialysis: case report and review of Oerskovia infections in humans. J Clin Microbiol.

[31] Reina J, Llompart I, Altés J (1991). An axillary abscess produced by Oerskovia turbata in an AIDS patient. Rev Clin Esp.

[32] Borra S, Kleinfeld M (1996). Peritonitis caused by Oerskovia xanthineolytica in a patient on chronic ambulatory peritoneal dialysis (CAPD). Am J Kidney Dis.

[33] Shah M, Gentile RC, McCormick SA, Rogers SH (1996). Oerskovia xanthineolytica keratitis. CLAO J.

[34] Harrington RD, Lewis CG, Aslanzadeh J, Stelmach P, Woolfrey AE (1996). Oerskovia xanthineolytica infection of a prosthetic joint: case report and review. J Clin Microbiol.

[35] Lujan-Zilbermann J, Jones D, DeVincenzo J (1999). Oerskovia xanthineolytica peritonitis: case report and review. Pediatr Infect Dis J.

[36] Espada FV, Costa PM, Da Cunha AL, Sarmento A, Mesquita Montes J, Cruz ME (2003). Spondylodiscitis. Acta Medica Port.

[37] Heym B, Gehanno P, Friocourt V, Bougnoux ME, Le Moal M, Husson C (2005). Molecular detection of Cellulosimicrobium cellulans as the etiological agent of a chronic tongue ulcer in a human immunodeficiency virus-positive patient. J Clin Microbiol.

[38] Yilmaz E, Ozakin C, Sinirtaş M, Evci C, Akalin H, Gedikoğlu S (2006). Meningitis caused by Oerskovia xanthineolytica. Mikrobiyol Bul.

[39] Thomas M, Padmini SB, Govindan VK, Appalaraju B (2007). Oerskovia turbata and Myroides species: rare isolates from a case of acalculus cholecystitis. Indian J Med Microbiol.

[40] Tucker JD, Montecino R, Winograd JM, Ferraro M, Michelow IC (2008). Pyogenic flexor tenosynovitis associated with Cellulosimicrobium cellulans. J Clin Microbiol.

[41] Haydushka IA, Martinez-Martinez L, Markova N, Genova SN, Kantardjiev TV, Valero-Guillen PL (2010). Fatal pneumonia in a newborn caused by Oerskoxia xanthineolytica. J Pediatr Infect Dis.

[42] Akçakaya AA, Sargin F, Erbil HH, Yazici S, Yaylali SA, Mesçi C (2011). A cluster of acute-onset postoperative endophthalmitis over a 1-month period: investigation of an outbreak caused by uncommon species. Br J Ophthalmol.

[43] Jaru-ampornpan Pimkwan, Agarwal Anita, Midha Narinder K., Kim Stephen J. (2011). Traumatic Endophthalmitis due toCellulosimicrobium cellulans. Case Reports in Ophthalmological Medicine.

[44] Kar S, Basu S, Sharma S, Das T (2011). Endogenous endophthalmitis caused by bacteria with unusual morphology in direct microscopic examination of the vitreous. Indian J Ophthalmol.

[45] Betancourt Castellanos L, Ponz Clemente E, Fontanals Aymerich D, Blasco Cabañas C, Marquina Parra D, Grau Pueyo C (2011). First case of peritoneal infection due to oerskovia turbata (Cellulosimicrobium funkei). Nefrologia..

[46] Magro-Checa C, Chaves-Chaparro L, Parra-Ruiz J, Peña-Monje A, Rosales-Alexander JL, Salvatierra J (2011). Septic arthritis due to Cellulosimicrobium cellulans. J Clin Microbiol.

[47] Sug Kim J, Won Lee T, Gyoo Ihm C, Jin Kim Y, Mi Moon S, Joo Lee H (2015). CAPD peritonitis caused by co-infection with Cellulosimicrobium cellulans (Oerskovia xanthineolytica) and Enterobacter cloacae: a case report and literature review. Intern Med.

